# A Delphi Exploration of Toileting Activity Performance in Individuals With Cerebral Palsy Within the ICF-CY Framework: Unveiling Influential Factors

**DOI:** 10.1155/2024/9994862

**Published:** 2024-10-07

**Authors:** Azade Riyahi, Malahat Akbarfahimi, Mehdi Rassafiani, Zahra Pournasiri, Maryam Ahmadi, Afsoon Hassani Mehraban

**Affiliations:** ^1^Rehabilitation Research Center, Department of Occupational Therapy, School of Rehabilitation Sciences, Iran University of Medical Sciences, Tehran, Iran; ^2^School of Allied Health, Exercise and Sports Sciences, Charles Sturt University, Albury, Australia; ^3^Pediatric Neurorehabilitation Research Center, The University of Social Welfare and Rehabilitation Sciences, Tehran, Iran; ^4^Pediatric Nephrology Research Center, Research Institute for Children's Health, Shahid Beheshti University of Medical Sciences, Tehran, Iran; ^5^Department of Health Information Management, School of Management and Medical Information Sciences, Iran University of Medical Sciences, Tehran, Iran

**Keywords:** activities of daily living, cerebral palsy, Delphi technique, functional status, International Classification of Functioning, Disability and Health, toileting

## Abstract

**Introduction:** Cerebral palsy (CP) is a common motor and postural developmental disability impacting daily activities like toileting. Despite its importance, toileting has often been undervalued in healthcare. This study is aimed at identifying and assessing the level of influence of various factors affecting toileting in individuals with CP using the International Classification of Functioning, Disability and Health for Children and Youth (ICF-CY) framework.

**Methods:** The Delphi method was employed to reach a consensus on factors influencing toileting in individuals with CP. One hundred fifty-eight professionals from 17 countries, each with at least 3 years' experience in CP and/or toileting, participated in this two-round study. Ethical approval (IR.IUMS.REC.1400.1111) and informed consent were obtained. The questionnaire, available in Farsi and English, used a Likert scale (5 = *very high impact* to 1 = *no impact*). Factors with 75% agreement and a mean above 3.75 were included in the final list, while those with 50%–75% agreement or a mean between 2.5 and 3.75 proceeded to the second round. Factors were initially identified through a literature review and research team consultation and categorized according to the ICF-CY framework using the ICF 10 RULES. Validation and refinement were done through focus groups with caregivers and experienced professionals to ensure alignment with the framework and methodological rigor.

**Results:** The study highlighted significant factors influencing toileting activities in individuals with CP. Within the ICF-CY framework, “neuromusculoskeletal and movement-related functions” scored highest at 94.5% in “body functions and structures.” “Toilet environment” scored 90.5% in “environmental factors.” Support and relationship factors scored 82.3%. Attitudes toward “menstruation” ranked highest at 92.9%. Associated deficits with CP ranked top in personal factors at 93.7%.

**Conclusion:** The study identifies key factors influencing toileting activity performance in individuals with CP. “Main caregiver” emerges as a pivotal factor, followed by neuromusculoskeletal functions, personal factors, attitudes toward menstruation, the toilet environment, and support and relationships.

**Summary**

• Address toileting comprehensively for effective implications by allied health professionals.
• Heighten awareness among medical teams, caregivers, and clients for timely interventions.
• Consider body function and structure factors such as neuromusculoskeletal and movement-related functions, sensory functions, and pain, as well as cognitive/perceptual functions to optimize management strategies.
• Include environmental factors related to the main caregiver, the physical environment of the toilet, support and relationships, and personal factors like CP-related deficits in teaching and supporting toileting activities in individuals with CP.
• Acknowledge attitudes and cultural differences to help healthcare professionals and caregivers provide more culturally sensitive and effective care for children and individuals with diverse toileting needs.

## 1. Introduction

Cerebral palsy (CP) is a common motor and postural developmental disability that begins in infancy and persists throughout the lifespan [1]. With an average incidence of 2–2.5 individuals per thousand births, CP poses significant challenges globally [2, 3]. CP is characterized by complex impairments in the central nervous system, leading to deficits in movement, sensation, perception, cognition, communication, and behavior. These impairments greatly impact activities of daily living (ADL) including toileting [1, 4, 5].

Toileting problems in individuals with CP could involve both urination and defecation. These individuals frequently encounter lower urinary tract dysfunctions like urinary retention, bladder incontinence, neurogenic bladder, and overactive bladder [6, 7]. Additionally, gastrointestinal disorders, including constipation and fecal incontinence, are prevalent [8]. These challenges, compounded by CP-associated impairments, pose substantial obstacles to effective toileting, leading to difficulties in participation in other daily activities [9, 10]. Inadequacies in toileting skills during childhood can lead to various medical problems, social isolation, and diminished quality of life [11]. Conversely, mastering toileting skills enhances self-confidence, physical comfort, independence, and social acceptance while alleviating the caregiver burden [12, 13].

Because toileting constitutes one of the most intimate and essential personal activities of daily living (PADL), demanding active engagement, and significantly contributing to overall well-being [11], it is necessary to investigate it thoroughly. Given the necessity of accounting for various aspects and identifying all effective factors for investigating each issue, it is imperative to focus on this area with a detailed examination of all elements that influence efficient toileting activities.

There are different definitions for toileting function, but within the International Classification of Functioning, Disability and Health (ICF) framework developed by the World Health Organization (WHO), which is agreed upon by all health professionals, it encompasses processes such as recognizing the need to defecate, planning elimination (including urination, defecation, and menstruation), and postelimination self-care [14, 15].

Toileting is a multifaceted human action involving motor, cognitive, sensory, communicative, and psychological aspects. It includes various stages, such as transferring, dressing, elimination, and hygiene [16–18]. The research underscores the influence of physiological, individual, environmental, cultural, and religious factors on the progression of toileting stages [19–21].

Despite its profound impact across various healthcare dimensions—including physical, psychosocial, spiritual, cultural, and even sexual and marital aspects [22–24]—toileting has often been marginalized in many societies. Sociocultural and religious factors divert attention from toileting-related disorders compared to other ADL domains, perpetuating negligence from medical staff, specialists, therapists, individuals, and caregivers alike [25, 26]. This oversight not only affects the well-being of individuals with CP but also exacerbates their challenges in performing toileting activities.

In spite of these insights, existing research inadequately explores the nuanced toileting needs of individuals with CP. The existing studies related to toileting are more focused on the body functions and structures and physiological aspects, which are more based on the medical model than on the functional aspects, as emphasized in the ICF model [27]. These studies often lack in-depth analyses of sociocultural, psychological, and environmental factors influencing toileting outcomes. Furthermore, there is a lack of consensus on effective therapeutic approaches integrating these multifaceted aspects within clinical practice.

The complexity of CP and the diversity of clinical manifestations, as defined in its latest characterization, underscore the necessity for a comprehensive assessment of functions [28, 29]. The ICF system provides a standardized language for healthcare professionals to comprehensively describe health issues, focusing on functional activities including toileting, while analyzing “body functions and structures” and “activities and participation” at personal and environmental levels [30, 31].

Therefore, this study is aimed at addressing these gaps by identifying and establishing a consensus on the factors influencing toileting activities in individuals with CP using a holistic approach grounded in the ICF-CY framework. Furthermore, the study seeks to determine the impact of these factors on toileting activity execution. By employing a Delphi method involving a diverse group of professionals—including CP specialists and caregivers—this research seeks comprehensive insights into the multifaceted challenges faced by individuals with CP in toileting activities.

## 2. Methods

### 2.1. Guideline Overview

This study adheres to the CREDES (Guidance on Conducting and REporting DElphi Studies) framework, which provides structured guidance for conducting and reporting Delphi studies. The CREDES checklist ensures methodological transparency and rigor throughout the research process. Key aspects include justifying the methodological choice, defining consensus criteria, preventing biases through rigorous survey instrument piloting, and transparently reporting results to illustrate consensus evolution [32].

### 2.2. Study Design

The Delphi methodology, a widely used approach in healthcare, was adopted to establish consensus on clinical matters [33]. The present Delphi study was conducted in two rounds and involved both national and international professionals, following the CREDES guide [32]. The Delphi technique, an iterative process, involves participants anonymously expressing their opinions to reach consensus through successive rounds [34].

Ethical approval for this study was obtained from the Research Ethics Committee of Iran University of Medical Sciences (IR.IUMS.REC.1400.1111). The approval ensures that the study meets ethical standards in research involving human participants.

### 2.3. Informed Consent Process

Participants in this study were provided with detailed information regarding the study's purpose, procedures, and potential risks and benefits. An email was sent to each participant containing the ethical code of conduct and an estimated completion time of approximately 25 min for the first round and 15 min for the second round of the Delphi survey. By voluntarily completing and submitting the survey, participants implied their informed consent to participate in the study.

The informed consent process included explicit information on confidentiality, voluntary participation, and the right to withdraw at any time without penalty. This approach aimed to ensure transparency and adherence to ethical standards throughout the research process. Measures to safeguard participant information included anonymizing data and secure storage of records.

### 2.4. Participants

Professionals working in the field of pediatric and adult disabilities, particularly CP and/or toileting function, were identified through clinical and academic settings. A professional in this study met specific criteria: (1) graduation from fields such as occupational therapy, physiotherapy, nursing, or psychology in basic sciences, or fields like neurology, urology, nephrology, gastroenterology, or nutrition within medical sciences. (2) Possession of a minimum of 3 years of experience practicing with children and adults with disabilities, including children with CP, or contributions to CP or toileting literature. By agreement with the authors, the threshold of requiring a minimum of 3 years of experience was established due to the availability of participants within this category. Additionally, beyond this timeframe, many individuals have encountered a critical mass of clients in their practice, contributing to the formation of an experience curve for these participants. (3) Professionals proficient in English or Persian were included, as the questionnaire was available in both languages to ensure accessibility for all potential participants.

Due to the limited number of professionals in the field, participants were identified by the snowball method, and availability was taken into consideration. The following routes were used for identification: (1) lead or responsible authors of articles with keywords related to toileting and/or CP, (2) clinicians with research interests connected to CP or toileting in associations and institutions including CanChild, (3) ResearchGate, and (4) LinkedIn. Professionals from various geographic regions were invited, encompassing Europe, North America, South America, Australia, Africa, Asia, and the Middle East. The snowball method facilitated participant recruitment based on availability and met the study's entry criteria. Potential biases were mitigated by verifying each participant against the specified inclusion criteria.

The sample size was determined based on established practices in Delphi methodology literature, which typically recommends panel sizes ranging from a minimum of 10 participants to a maximum of 1000 participants. However, practical considerations such as data management and logistical challenges associated with multiple survey rounds suggest that panels larger than three digits are uncommon. Optimal panel sizes for concluding rounds generally range from 30 to 50 participants, ensuring a balance between diversity of perspectives and manageable consensus building [35]. A total of 880 professionals were identified and sent invitations to participate. Ultimately, 134 professionals from Iran and 24 from other countries participated. The opinions of 158 professionals were utilized in the first Delphi round, and 127 professionals were involved in the second round to achieve saturation, as recommended by the consensus-based standards for the selection of health measurement instruments [36]. A larger sample size enhances Delphi's study quality by reducing group error and bolstering external validity, a principle applied in this study [37].

### 2.5. Delphi Methodology

#### 2.5.1. Delphi Questionnaire Preparation

**Figure 1:**
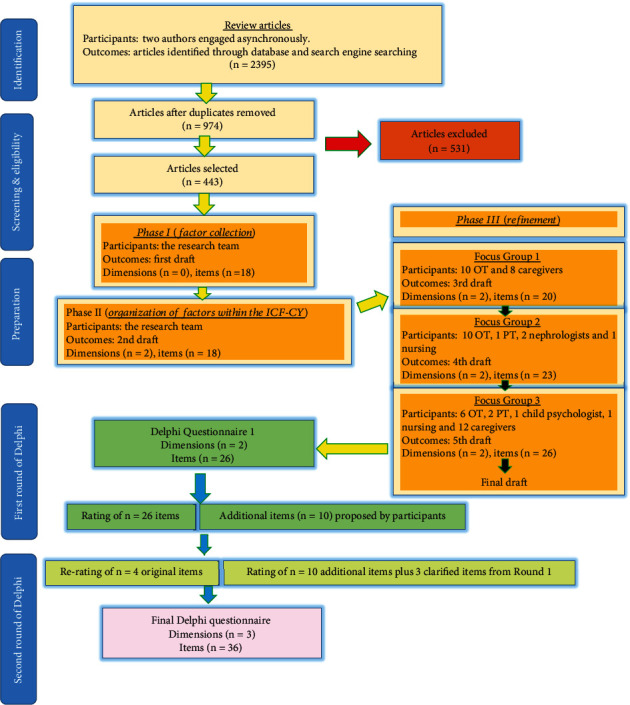
Flowchart depicting the process of crafting the Delphi questionnaire and the two successive survey rounds of the Delphi study.

The questionnaire is a crucial tool for obtaining professional opinions in the Delphi method. The present study developed a questionnaire concerning factors influencing the toileting activity stages of individuals with CP. This process comprised three phases.  Figure 1 depicts various stages and phases of this Delphi study.

• Phase I (factor collection): Articles with keywords related to the study's focus (related to toileting or factors influencing toileting activities) were sourced between 1990 and 2022 in both English and Farsi. The search strategy and criteria for article inclusion are detailed in the accompanying documentation (Supporting Information 1). Initially, factors were identified through a review of the literature. Factors influencing toileting were extracted from these articles based on their relevance. Then, a list of potentially influential factors on toileting activity implementation was assembled. This list underwent review in multiple sessions with the research team, incorporating team insights. Subsequently, the “first draft version of the questionnaire” was established (for further information, please see Figure 1).
• Phase II (organization of factors within the ICF-CY framework): In this phase, for comprehensive structuring, the collected influential factors were organized into the ICF-CY framework using the ICF 10 RULES, and each factor was categorized in the relevant component based on its concept by Stucki [38]. This resulted in the “second draft version of the questionnaire” (Figure 1).
• Phase III (refinement): In the third phase, identified factors were refined and supplemented through discussions in three separate focus groups involving a total of 34 experienced professionals and 20 caregivers of individuals with CP. During this phase, participants reviewed and validated the identified factors. Factors achieving 75% agreement or higher were systematically integrated into the questionnaire for subsequent rounds. New factors proposed during these discussions were also considered and incorporated into the appropriate sections of the ICF framework after review by the research team. The revised draft entered the next session, leading to the “final version of the questionnaire” (no factors were eliminated due to the high levels of agreement achieved during this validation process). These sessions allowed for the comprehensive exploration and validation of the factors identified in the literature review phase, ensuring that all relevant aspects were considered. The focus group participants included experienced professionals with at least 3 years of relevant work experience, along with caregivers directly involved in the care of individuals with CP. Demographic information of the participants in each session as well as the changes in the draft version of the questionnaire after each session are shown in Figure 1.

The questionnaire, available in both Farsi and English, was distributed to participants via email in their respective languages. To ensure consistency in meaning and understanding across languages, the Farsi questionnaire was initially translated into English by a bilingual native speaker and then meticulously reviewed and edited by the research team. Additionally, a second translator conducted a further review to guarantee accuracy and alignment between the two versions.

Demographic information was collected, followed by a Likert scale rating for each factor ranging from 5 = *very high impact* to 1 = *no impact*. Participants could suggest additional factors.

### 2.6. Delphi Survey

#### 2.6.1. Round Procedures

During each round of the Delphi study, the progression of questionnaire items was guided by established Delphi principles. Specifically, items were either confirmed for retention, modified based on feedback, or removed based on predefined thresholds of consensus. These decisions were informed by both the percentage of agreement (75% or above) among participants and the mean ratings (3.75 or above) assigned to each item. This approach aligns with standard Delphi methodology, ensuring that only the most relevant and supported factors progress through subsequent rounds. The specific thresholds for consensus and decision-making processes were chosen to maintain rigor and to systematically refine the questionnaire based on the input from experienced professionals [39–41].

#### 2.6.2. First Round

The final questionnaire was sent via email to national and international professionals for the first Delphi round, lasting 4 weeks for Iran and 8 weeks for other countries, factoring in the New Year holidays. Reminder emails after 2 weeks were sent as needed. After collecting 134 responses from Iran and 24 from other countries, the first round was completed. Items with an agreement percentage of 75% or above and a mean surpassing 3.75 moved forward, along with items added by participants and those with agreement or influence ratings between 50% and 75% and/or a mean between 2.5 and 3.75.

#### 2.6.3. Second Round

A new questionnaire was constructed for the second round, presenting values from the first round for items not meeting predetermined agreement levels. Participants received this questionnaire along with any newly suggested factors, providing their opinions via a 5-point Likert scale. After receiving 110 responses from Iran and 17 from other countries, the second round ended.

### 2.7. Data Analysis and Consensus Criteria

Items from the first round were considered for inclusion in subsequent rounds based on specific consensus criteria. Factors achieving an agreement percentage of 75% or higher among participants, coupled with a mean score exceeding 3.75 on the Likert scale, were retained. This approach is supported by the literature, where a 75% agreement threshold is widely used in Delphi studies to indicate strong consensus among experts. The mean score criteria ensure that retained factors are rated highly in terms of their importance or relevance, capturing the intensity of consensus [39, 40, 42–44]. Factors with moderate agreement, falling within the range of 50%–75% agreement and/or mean scores between 2.5 and 3.75, were carried forward to the second round for further deliberation. This approach allows for the exploration and refinement of factors with moderate support. Items that did not meet these consensus thresholds—agreement percentages below 50% and/or mean scores below 2.5—were excluded from subsequent rounds, maintaining methodological rigor and focusing on factors with higher levels of agreement and perceived importance [41, 45, 46] (Figure 1).

To manage conflicting opinions or discrepancies among participants, several procedures were implemented. After each round, detailed feedback reports summarizing areas of agreement and disagreement were shared anonymously. Participants provided justifications for their responses, which were also shared anonymously to help understand differing viewpoints. The iterative nature of the Delphi method allowed participants to reconsider their initial responses based on aggregated feedback. Consensus thresholds were set, retaining factors with 75% or higher agreement and mean scores above 3.75, while factors with 50%–75% agreement and/or mean scores between 2.5 and 3.75 were further deliberated. Items below these thresholds were excluded. This approach ensured a systematic resolution of discrepancies.

### 2.8. Cultural Taboos and Terminology Adjustments

To address cultural taboos in toileting practices, the study began with a comprehensive literature review, examining peer-reviewed articles, cultural studies, and international health guidelines. Insights were further refined through an expert panel with diverse cultural backgrounds and focus groups involving caregivers and healthcare providers from various contexts. During the Delphi process, participants were given opportunities to flag and provide feedback on culturally sensitive items. Adjustments were made to ensure respect for cultural norms, such as privacy and modesty, and these revised items were validated in subsequent rounds to confirm their acceptance across different cultural perspectives. This iterative process ensured that the final consensus reflected a broad and respectful understanding of cultural considerations in toileting practices.

Additionally, the significance of language and cultural sensitivity, especially for sensitive topics like toileting practices, was addressed by revising clinical and technical language based on feedback from a diverse panel of participants. Culturally specific practices and terminologies were incorporated to align with varied cultural norms, enhancing the study's sensitivity and appropriateness across different cultural contexts [47, 48].

## 3. Results

### 3.1. Characteristics of Delphi Participants

A total of 158 professionals from 17 countries participated in this Delphi consensus study. Among the 158 participants who completed the first round, 127 successfully concluded the second round. Notably, the detailed characteristics of the participants are outlined in Table 1.

### 3.2. Delphi Consensus Process

#### 3.2.1. First Round

During the initial round, consensus was achieved regarding the effectiveness and impact levels (rated as very high and high impact) of 22 presented factors. Based on predetermined agreement criteria, these factors were incorporated into the final list of factors that influence the implementation of toileting activities. However, for four factors (voice and speech functions, attitudes on the implementation of health and hygiene practices, attitudes on the way the clothing management is executed, and attitudes on issues related to menstruation), consensus was not achieved based on predetermined agreement criteria and carried out to the second round (Table 2). The compiled data are presented in Supporting Information 2.

#### 3.2.2. Second Round

**Figure 2:**
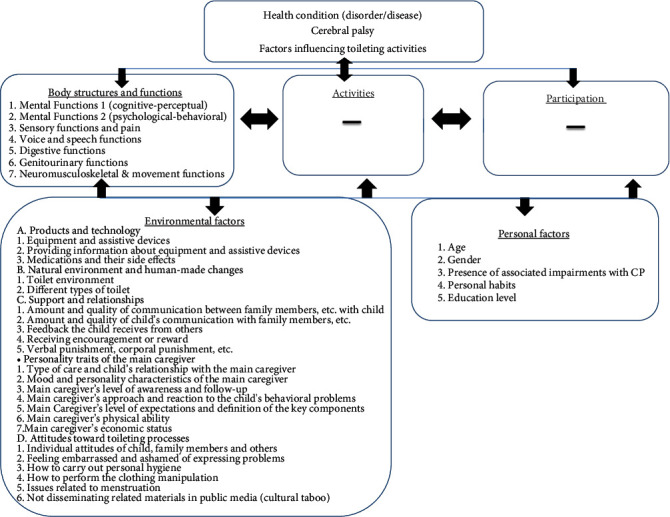
Factors impacting toileting activities in cerebral palsy based on ICF-CY.

In the second round, four initially inconclusive factors were revisited and modified based on professional input. Additionally, sensory functions, motor functions, and the third caregiver characteristic (caregiver's level of cognition and awareness) were reintroduced, considering insights from the previous round and subsequent refinements. Participants provided feedback on these factors, along with 10 other proposed effective factors, all of which gained approval and were integrated into the final list in alignment with the ICF-CY model (Figure 2). The compiled data are presented in Supporting Information 3.

In the initial round, 80.3% of professionals recognized the substantial impact of sensory functions. Following professional feedback, the item underwent refinement, achieving an impressive 92.9% consensus threshold in the second round. Pain, specifically addressing its effect on toileting function, also gained substantial endorsement at 85.1%. It is noteworthy that pain was added by the professionals in the first round and proceeded to the second round for polling.

Consistent with previous investigations, the study revealed that in the initial round, 84.1% of professionals recognized the significance of neuromusculoskeletal and movement-related functions. Subsequently, incorporating professional insights and refinements, which included aspects such as motor control, coordination, postural, and praxis control, the study achieved a consensus of 94.5% in the second round.

Upon further input from professionals, items related to the main caregiver's level of cognition and awareness about the child's readiness for toileting and generalization of strategies to home environments, along with two new proposed items—namely, the main caregiver's economic status and physical ability—achieved consensus scores of 96.0%, 91.3%, and 90.5%, respectively, in the second round.

In the initial round, 50.6% of professionals acknowledged the significant impact of voice and speech functions. Following refinement, this recognition increased to 75.6%.

During the initial round, three attitudes—personal hygiene, clothing manipulation, and menstruation—were rejected. Additionally, a new item related to the nondissemination of materials in public media was introduced following suggestions from participants and included for evaluation in the second round. In the subsequent round, the impact of the items ranged from 86.6% to 92.9%. Attitudes related to menstruation obtained the highest percentage (92.9%), followed by attitudes concerning personal hygiene, clothing manipulation, and nondissemination of related materials in public media, all with an equal agreement level of 86.6%. The remaining items achieved an agreement level of 81.6%.

It is necessary to explain that the other new items suggested by professionals at the end of the first round and agreed upon in the second round included the consumed medications and their side effects, as well as personal factors such as age, gender, personal habits, presence of diseases associated with CP, and level of education. The compiled data are presented in Table 2.

### 3.3. Explanation for Nonconsensus Factors and Detail on Modifications

Four factors initially deemed inconclusive were refined to achieve consensus. Feedback indicated the original description of voice and speech functions was too broad and lacked clarity, so it was revised to focus on how these functions impact toileting activities, such as communicating the need to use the toilet. Attitudes toward personal hygiene practices were split into two separate factors to clarify distinct issues and address the conflation of beliefs and practices related to hygiene. The description of attitudes toward clothing manipulation was seen as too abstract and was clarified with specific examples to better reflect its impact on toileting. The scope of attitudes related to menstruation was narrowed to focus on a specific subdimension relevant to toileting, addressing concerns that it was too broad and overlapping with other factors. These changes were made to enhance clarity, relevance, and precision based on panel feedback.

### 3.4. Ranking of Factors Influencing Toileting Execution: From Most to Least Influential According to the ICF Components

#### 3.4.1. Body Function and Structure Factors

**Figure 3:**
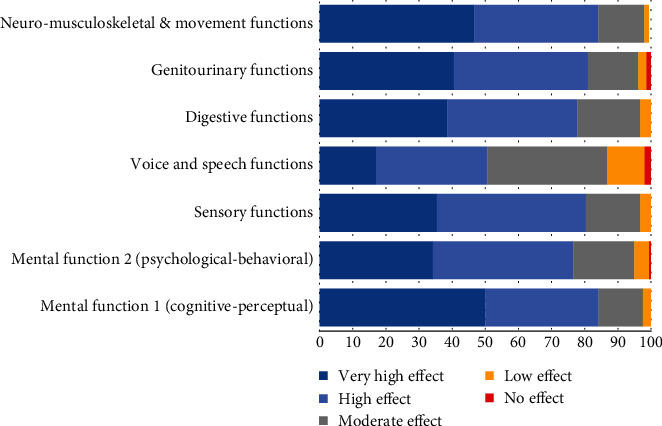
Agreement regarding the effectiveness of factors associated with body functions and structures.

In this study, neuromusculoskeletal and movement-related functions were identified as the most influential body functions impacting toileting execution, followed by sensory functions and pain. Cognitive/perceptual functions ranked fourth, while urinary functions were ranked fifth. Digestive system functions were positioned sixth, with psychological/behavioral functions ranking seventh and voice and speech functions ranking eighth and last in influencing toileting execution. It should be noted that the ICF model divides mental functions into global and specific mental functions. This study has used cognitive/perceptual and psychological/behavioral functions based on themes identified from the literature review and focus groups (Figure 3).

#### 3.4.2. Environmental and Technological Factors

Among environmental factors within the realm of production and technology-related factors, the consumed medications and their side effects on toileting function garnered the highest consensus, followed by equipment and assistive devices used for toileting function and providing information about these devices. The toilet environment was rated as the most influential natural environmental factor, with toilet type following closely behind.

#### 3.4.3. Support and Relationship Factors

Communication quality and feedback on toileting execution were recognized as primary influencers in support and relationships. Communication among family members with the child/adolescent and the consequences of improper toileting also significantly impacted toileting outcomes. Additionally, receiving encouragement or reward for error prevention during toileting ranked third in this domain.

#### 3.4.4. Main Caregiver Factors

After the conclusion of the second round, the most influential factor to the least influential factor, in order, are the caregiver's level of cognition and awareness about the child's readiness for toileting and generalization of strategies to home environments; the caregiver's approach and reaction to the child's behavioral problems; the caregiver's economic status; the caregiver's physical ability, mood and personality characteristics, type of care, and child's/adolescent's relationship with the caregiver; and the caregiver's level of expectations regarding toileting performance and his/her assessment of key aspects such as independence, safety, and efficiency.

#### 3.4.5. Attitude Factors

**Figure 4:**
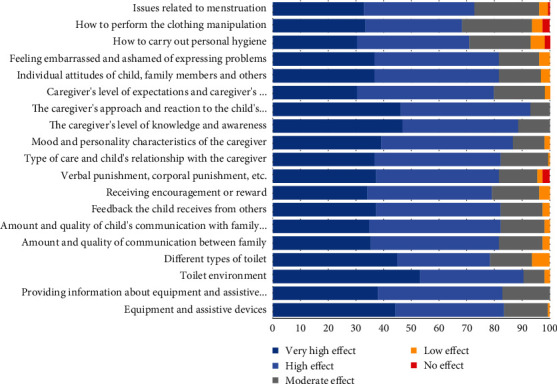
Agreement regarding the effectiveness of factors associated with the environment.

Attitudes related to menstruation were ranked as the most influential factors affecting toileting activity performance, followed by attitudes toward personal hygiene, clothing management, and public information about toilet training. Individual attitudes of children/adolescents, family members, and others about toileting, along with feelings of embarrassment or shame related to toileting and menstruation, held the third rank (Figure 4).

#### 3.4.6. Personal Factors

**Figure 5:**
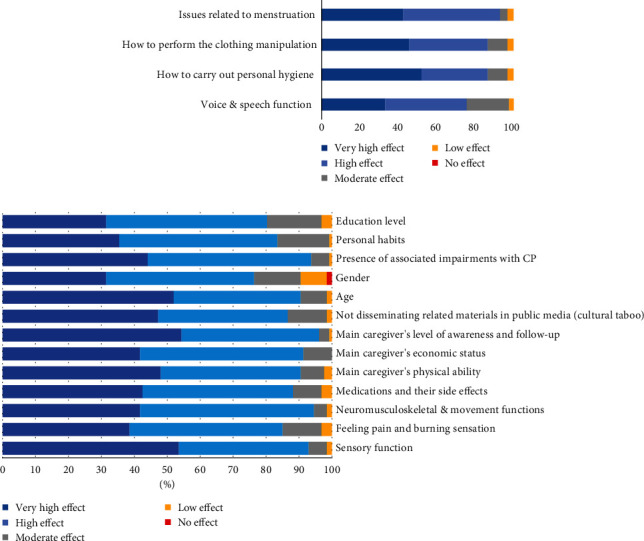
Agreement regarding the effectiveness of factors in Round 2 (body functions/attitudes/other factors).

Associated conditions were recognized as the primary influential personal factor affecting toileting activity performance, followed by age, personal habits, level of education, and gender (Figure 5).

## 4. Discussion

The aim of this study was to achieve a consensus about the factors influencing the multifaceted task of toileting within the context of a prevalent and complex health condition such as CP. The uniqueness of this study lies in its comprehensive exploration and examination of the multitude of factors influencing toileting activities and gauging the extent of their effects through the perspectives of diverse healthcare professionals, including physicians, therapists, nurses, and psychologists from various cultural backgrounds. Also, we systematically identified and organized all of these factors, using the ICF model as a guiding framework, across various domains such as body functions and structures, personal, and environmental factors. The identified factors are discussed in the following, respectively.

In the context of *body functions and structures* related to toileting activity, this study specifically delves into mental functions (cognitive/perceptual and psychological/behavioral functions); sensory functions and pain; voice and speech functions; functions of the digestive, metabolic, and endocrine systems; genitourinary and reproductive functions; and neuromusculoskeletal and movement-related functions. These aspects have been discussed in detail in subsequent sections, shedding light on their significant roles in shaping the toileting experiences of individuals with CP.

This two-round Delphi study revealed significant professional consensus on the impact of mental functions, emphasizing the roles of *cognitive/perceptual* and *psychological/behavioral* functions on toileting activities. Cognitive-perceptual and psychosocial functions are crucial in toileting, as highlighted by Baird, Bybel, and Kowalski and Harris [49, 50]. Ölçer and Cal and Matson et al. emphasized the role of cognitive development, particularly language skills, in effective toilet training [51, 52]. Cognitive impairments in individuals with CP complicate toileting activities [9, 53, 54]. Similarly, Ozcan and Cavkaytar noted the significant influence of psychosocial factors on toileting success in children with CP [55]. Wagner, Niemczyk, and von Gontard identified toilet refusal syndrome (TRS) and toilet phobia, both presenting with physical and behavioral issues, as common problems in preschool children [56]. These findings collectively highlight the importance of cognitive and psychosocial factors in the toileting process for individuals with CP, which should be carefully considered for implications.

The present study highlighted the significant role of *sensory functions* in toileting activities. *Pain* and burning sensations before, during, or after urination and defecation were also deemed important. Research by others supports these findings [57]. Ruffini et al. found sensory processing issues hinder the recognition of the need to toilet [58]. Yip, Powers, and Kuo noted that pain during toileting could lead to avoidance behaviors, exacerbating toileting challenges [59]. Baker-Malone showed that children with CP often experience heightened pain and sensory sensitivity, complicating toileting [60]. These findings emphasize the influence of sensory and pain factors in toileting activities, providing a better understanding of the challenges faced by individuals with CP.

Participants emphasized the significant impact of *voice and speech functions* on toileting execution in this study. Effective communication is essential for signaling the need for assistance or conveying specific requirements during toileting [61, 62]. Many children with CP exhibit delayed or disordered speech, with 33%–63% experiencing difficulties, including anarthria [63]. For those with speech impediments, alternative communication methods, such as gestures or signs, are often used to convey toileting needs [18, 64]. Both verbal and nonverbal communication methods require specific mental functions related to language, which are particularly challenging for individuals with CP [64, 65]. Based on this Delphi survey and existing literature, these functions should be considered when addressing toileting activity for individuals with CP.

In this study, participants agreed on the significant impact of *digestive system* factors on toileting. The research underscores the digestive system's pivotal role in stool elimination during toileting activities [9, 66–68]. Factors such as diet, fluid intake, mobility levels, medication use, and side effects impact the functionality of this system in individuals with CP [8, 69]. Limited mobility often leads to constipation, affecting 26%–90% of children with CP [9, 70]. Delays in achieving bowel control and high constipation prevalence are well documented, highlighting the complexity of digestive issues in children with CP [67, 71, 72]. This study reinforces the importance of addressing digestive system factors in managing toileting, aligning with existing literature and emphasizing the need for comprehensive approaches to mitigate these challenges.

Consensus was obtained in this study on the significant impact of *urinary functions* on toileting. Toileting involves intricate urinary mechanisms, particularly bladder emptying [73]. Many children with CP experience urinary disruptions due to conditions like neurogenic bladder, heightened detrusor muscle activity, urinary tract infections, and delayed urinary control [7, 74]. A study in 2017 found that 20.5% of individuals with CP reported urinary incontinence, 38.5% had nocturnal enuresis, 48.7% faced urinary urgency, and 36.8% experienced urge urinary incontinence [22]. Participants in this study, consistent with previous research, recognized the substantial impact of these urinary dysfunctions on toileting activities.

*Neuromusculoskeletal and movement-related functions* were recognized as crucial factors in the different stages of toileting activities in this Delphi survey. Proficient motor skills are essential for children to independently manage daily tasks, including toileting [75]. These skills are particularly vital following the onset of CP, where impairments restrict tasks such as reaching the toilet room, managing clothing, sitting comfortably, maintaining balance, and participating in posttoileting hygiene [16, 76]. This study solidifies these functions as pivotal in toileting execution, emphasizing the need for special attention to these functions.

Among the *environmental factors*, the dimensions of products and technology, the natural environment and human-made changes, support and relationships, the main caregiver, and attitudes will be discussed separately.

This study reached a consensus on the significance of *products and technology* and *the natural environment and human-made changes* in toileting activities. The toilet environment—including toilet type, assistive equipment, and aids—plays a crucial role in enabling safe and independent toileting [77]. Research highlights that for individuals with CP, environmental adaptations like changing the toilet type or using assistive devices can enhance toileting performance and independence [19, 78–80]. Østensjø, Carlberg, and Vøllestad and De-Rosende-Celeiro et al. emphasize the importance of assistive products and environmental modifications in improving functional independence and reducing care demands [79, 81]. Rigby, Ryan, and Campbell noted improved self-care in children with CP using adaptive seating at home [82]. Additionally, Liu et al. suggest adapting environments to individuals' capabilities, such as promoting shower facilities and pedestal pans [83]. Netto et al. recommend using toilet seat reducers or potty chairs to improve security and comfort during toileting [84]. Overall, this study supports the significant role of environmental and technological factors in effective toileting and underscores the need for considering environmental strategies.

Regarding the *support and relationship* components, this study explored dimensions related to the interpersonal relationships of children/adolescents with CP. These factors were pivotal in shaping the toileting experiences and outcomes. Other research also underscores the importance of these factors. Mota and Barros discussed how parental attitudes and expectations impact child development and behavior, advocating for realistic expectations to improve parent–child interactions and developmental outcomes [85]. Gunay and Oltuluoglu found that punitive maternal attitudes during toilet training led to psychological strain and resistance in children, while supportive approaches promoted better developmental outcomes and emotional well-being [86]. Koc et al. similarly noted that mothers with lower education often use punitive methods such as forcing and threatening the child, which can cause discouragement and power struggles, whereas positive approaches lead to better outcomes [87]. These findings emphasize the importance of positive interpersonal relationships and support in shaping toileting experiences for individuals with CP, highlighting the need for fostering positive interactions.

Results from interviews and discussions underscored the pivotal role of the *main caregiver*, responsible for educating self-care activities, including toileting, given their enhanced familiarity with the child's conditions. Given the complexity of toileting performance, a dedicated section was allocated to exploring dimensions related to the main caregiver. This aimed to highlight their role in teaching toileting activities, especially when a different caregiver, such as a grandparent or nurse, assumes this role due to parental work commitments [86, 88, 89].

Consistent with these caregiver dynamics, Albaramki, Allawama, and Yousef identified familial support and maternal educational level as factors delaying toilet training initiation, illustrating the significant influence of caregiver characteristics on training outcomes [90]. Netto et al. discussed how parental work status influenced the age of toilet training initiation, emphasizing parental availability and involvement as critical factors [84]. However, other studies have not found a correlation between toilet training and the mother's employment status [91, 92] or the family's socioeconomic status [92]. Furthermore, Ölçer and Cal explored how parental personalities shaped toilet training outcomes, suggesting that positive caregiver traits could enhance the training process and subsequent child development [51]. In line with the present Delphi study, these studies underscored the multifaceted role of caregivers in shaping toileting behaviors and emphasized the need for tailored programs that considered caregiver dynamics to optimize child outcomes.

Another factor highlighted in this study is attitude. *Attitudes* play a crucial role in shaping private and culturally sensitive topics such as toileting and menstrual hygiene. Cultural attitudes and disparities lead to delays in toileting education and the persistent use of diapers in some families, reflecting broader patterns noted in other studies [61, 93–95]. Othman and Buys highlighted that while toileting and hygiene topics are often considered taboo, they are crucial for health and hygiene [96]. Hence, it is essential to acknowledge that each country has its own social norms and cultural standards regarding toileting practices, and each individual possesses their own unique habits, values, and routines [97, 98]. Mota and Barros highlighted the complexity of toilet training, which varies by culture and social norms, involving both physical and sociocultural readiness [85]. Moreover, Ölçer and Cal highlighted how parental attitudes during toilet training in childhood can influence both toilet habits and personality traits in adulthood, underscoring the long-term impacts of cultural and familial practices [51]. Gunay and Oltuluoglu identified cultural context as a key factor influencing the success of toilet training [86]. In conclusion, understanding and respecting diverse cultural attitudes and norms surrounding toileting practices are essential issues for developing strategies that align with individual and community beliefs.

Within the realm of environmental factors associated with products and technology, the impact of *medications and their side effects* on toileting function in individuals with CP has been recognized in the present study. Nearly all children with CP have additional disabilities due to CNS damage, managed through various medications and therapies [99]. The use of certain medications and their resultant side effects, including those related to antispasmodics and salivary medications, can lead to urinary and/or fecal problems, constipation, and so on, which ultimately affect toileting activities, which has been mentioned in previous studies. Tsakiris, Oelke, and Michel highlighted that drug-induced urinary incontinence can occur due to adverse effects on factors crucial for urinary continence [100]. Similarly, Wald discussed how common medications can contribute to chronic constipation, either as a primary effect or as a side effect of treatment, impacting bowel function negatively [101] and requiring fiber or laxatives [102]. These findings emphasize the need to consider the impact of medication side effects when investigating environmental factors affecting toileting in individuals with CP.

With reference to the viewpoints of the participants in the first round of this study, *personal factors* including age, gender, associated impairments, personal habits, and the individual's level of education have been discussed separately.

Among personal factors, a consensus was reached on *age* as an effective factor in this study. Sun and Rugolotto noted that age is a pivotal factor in toilet training, with the optimal age suggested between 2 and 4 years [103]. Commencing training after 18 months, when collaborative efforts with caregivers become more effective, is generally advised. Cultural variations exist, with toilet training practices starting from a few weeks after birth in some African tribes to later ages in the United States and Europe [91, 104, 105]. Achieving toilet training around the age of three is a significant developmental milestone for all children. However, children with CP may face challenges, potentially leading to delays in the training process [106]. While Mota and Barros highlighted various factors including age, at the start of training, Wyndaele et al. found that developmental signs, rather than age alone, are crucial for determining the right time to start toilet training and predicting its success in healthy toddlers [85, 107]. This Delphi study's findings reinforce the significance of age in toileting training, aligning with existing research on cultural and age-related variations in training practices.

The findings of this study highlighted the importance of *gender* as the influential personal factor affecting toileting activity performance. Research indicates that gender-specific anatomical and physiological factors impact toileting behavior and training [108, 109]. Studies have shown that girls tend to complete toilet training earlier than boys, potentially due to differences in anatomical and physiological factors, social expectations, and parenting practices [21, 91, 110]. This Delphi study's findings align with existing research on gender differences in toileting training and behavior, emphasizing the need for gender-specific approaches to training and support.

In this study, allied health professionals agreed on the significant impact of *associated impairments* on toileting. In cases of CP, beyond the physical, cognitive, and psychosocial impairments arising from the condition, the existence of associated impairments, including seizures, communication and speech impediments, auditory and visual complications, sluggish growth, irregular respiration, gastrointestinal symptoms, mastication and swallowing difficulties, nutritional concerns, and bowel and bladder issues, could further influence the toileting in individuals with CP [1, 54, 70, 111]. Given the intricate nature of toileting activity, its multifaceted requirements, and the challenges posed by these associated impairments, there is a critical need to investigate and manage them effectively.

This study also highlighted that specific patterns and *personal habits*, such as nutritional habits and urination and defecation habits, could engender diverse gastrointestinal, evacuative, and urinary issues or vice versa, ultimately influencing the quality of toileting activity performance [112]. A modified diet for individuals with CP that reduces fiber intake may cause constipation, leading to pain, anxiety, and complications like liquid stool leakage, UTIs, or urinary incontinence [113]. Chung et al. found that delayed bowel control, urinary tract infections, low paternal education, dual-income households, and lower income adversely affect urination and bowel habits in children with CP [112]. Mota and Barros stressed the importance of correct bladder and bowel voiding habits for maintaining a healthy life and good self-esteem, highlighting that urination and evacuation problems can cause significant discomfort for children and their families, impacting socialization and leisure activities [85]. The consistent findings across studies suggest that professionals attributed a substantial impact to these personal habits.

In this study, another effective personal factor for individuals with CP was the *level of education*. A segment of toileting training methods encompassed the utilization of books, instructions, brochures, illustrated materials, and so on, necessitating individuals to possess reading, writing, and comprehension abilities to effectively deliver such training. In essence, for individuals of school age and above, their level of education significantly influenced the provision, quality, acceleration, and success of toilet training [12, 49, 114, 115]. Netto et al. emphasized the importance of educational methods in toilet training, highlighting how they contribute to the developmental process. They noted that toilet training is a crucial developmental milestone influenced by a child's growth and maturation. Moreover, they pointed out that children born prematurely often experience delays in achieving toilet training due to associated motor, cognitive, academic, language, and behavioral challenges [84]. Understanding these factors may be effective in improving toilet training processes and supporting better developmental progress.

## 5. Conclusion

In alignment with the ICF-CY framework, this study identifies and ranks various factors influencing toileting performance. The leading factor is the domain of the main caregiver role, including their cognition, awareness of the child's readiness, teaching methods, and follow-up on toileting performance. Second in importance are “neuromusculoskeletal and movement-related functions” within the body functions and structures category. Personal factors, particularly “associated deficits with CP,” rank third. Attitudes related to “menstruation” are ranked fourth, followed by the “toilet environment” at fifth. The sixth rank is shared by “the amount and quality of the child's/adolescent's communication with family members” and “feedback received from family members regarding toileting steps” in the support and communication domain. Given the complexity of toileting, this study, using the ICF model, addresses factors within the components of body functions and structures, as well as personal and environmental domains. However, further research is needed to explore how these factors impact different stages of toileting and how to use them effectively for individuals with CP.

### 5.1. Recommendations

Based on this study, research recommendations include conducting evidence-based studies to evaluate the effect sizes of various factors identified in this study, assessing both statistical and clinical significance. This approach will help refine our understanding of how specific factors within the domains of body functions and personal and environmental factors influence toileting performance. By enhancing our knowledge in these areas, we can improve the development and implementation of effective toileting evaluations and interventions. Future research should focus on investigating the success rates of different toileting training methods for individuals with CP.

For clinical and practical recommendations, it is crucial to increase awareness among the toileting management team about the significant roles of factors identified in this study, such as caregiver cognition, neuromusculoskeletal function, and personal and environmental factors. Implementing compensatory strategies, using assistive devices, and making environmental modifications can enhance the toileting experience. Additionally, optimizing the physical environment of the toilet, including the selection of appropriate equipment, will support effective toileting and improve the quality of life for individuals. Addressing cultural and religious attitudes toward toileting and menstrual hygiene management and managing any extreme thoughts or attitudes will also be important for improving practical outcomes.

### 5.2. Limitations and Suggestions

This study faced limitations, including a lower number of international responses compared to national ones, which impacts generalizability. Also, recruitment was limited to caregivers due to restricted access to individuals with CP, affecting the depth of understanding of their perspectives. It is recommended to consider these in future research, which in turn would enhance the understanding of factors within each domain and their specific contributions to toileting activity performance.

## Figures and Tables

**Table 1:** Demographic characteristics of Delphi participants.

**First round of Delphi**							**Second round of Delphi**						
**Categorical variables**							**Categorical variables**						
**Participants**	**N** ** (%)**	**Gender**		**Education level**			**Participants**	**N** ** (%)**	**Gender**		**Education level**		
		**F**	**M**						**F**	**M**			
Occupational therapist	67 (42.4)	42	25	12 BSc	27 MSc	28 PhD	Occupational therapist	55 (43.3)	33	22	12 BSc	21 MSc	22 PhD
Physical therapist	19 (12.0)	14	5	6 BSc	4 MSc	9 PhD	Physical therapist	16 (12.6)	11	5	6 BSc	3 MSc	7 PhD
Pediatric nursing	15 (9.5)	13	2	11 BSc	2 MSc	2 PhD	Pediatric nursing	15 (11.8)	13	2	11 BSc	2 MSc	2 PhD
Clinical psychologist	12 (7.6)	7	5	2 BSc	7 MSc	2 PhD	Clinical psychologist	11 (8.7)	6	5	2 BSc	7 MSc	2 PhD
Pediatrician	10 (6.3)	10	0		9 S	1 SubS	Pediatrician	6 (4.7)	6	0		6 S	
Pediatric nephrologist	10 (6.3)	6	4		1 S	9 SubS	Pediatric nephrologist	7 (5.5)	4	3		1 PhD	6 SubS
Health and social development specialist	3 (1.9)	2	1			3 PhD	Health and social development specialist	2 (1.6)	2	0			2 PhD
Pediatric kidney specialist	3 (1.9)	3	0			3 SubS	Pediatric neurologist	2 (1.6)	2	0			2 SubS
Pediatric neurologist	2 (1.3)	2	0			2 SubS	Cognitive neuroscientist	1 (0.8)	0	1			1 PhD
Cognitive neuroscientist	2 (1.3)	0	2			2 PhD	Rehabilitation science specialist	1 (0.8)	1	0			1 PhD
Rehabilitation science specialist	2 (1.3)	1	1			2 PhD	Pediatric endocrinologist	1 (0.8)	1	0			1 SubS
Pediatric endocrinologist	1 (0.6)	1	0			1 SubS	Pediatric gastroenterologist	3 (2.4)	2	1		2 S	1 SubS
							Pediatric urologist	2 (1.6)	0	2			2 SubS
Pediatric gastroenterologist	3 (1.9)	2	1		2 S	1 SubS	Pediatric gastrologist	3 (2.4)	2	1		2 S	1 SubS
Pediatric urologist	4 (2.5)	3	1		1 DNP	3 SubS	Child and adolescent psychiatrist	1 (0.8)	1	0			1 SubS
Pediatric gastrologist	3 (1.9)	0	3		2 S	1 SubS	Speech therapist—Linguistics	1 (0.8)	1	0		1 MSc	
Child and adolescent psychiatrist	1 (0.6)	1	0			1 SubS	Total	127	85 (66.93)	42 (33.07)			
Speech therapist—Linguistics	1 (0.6)	1	0		1 MSc								
Total	158	108 **(68.35)**	50 **(31.64)**			

Continuous variables (mean ± SD)							Continuous variables (mean ± SD)						
Age		41.45 ± 11.09					Age		40.36 ± 10.86				
Educational activities, years		8.17 ± 9.62					Educational activities, years		7.07 ± 9.32				
Clinical activities, years		15.54 ± 9.18					Clinical activities, years		14.51 ± 8.79				
Executive activities, years		4.05 ± 6.43					Executive activities, years		3.68 ± 6.16				
CP work experience, years		7.94 ± 8.06					CP work experience, years		7.36 ± 7.36				

Country							Country						
Iran: 134; United States: 4; Canada: 4; Norway: 2; Turkey: 2; Brazil: 1; United Arab Emirates: 1; Kuwait: 1; Ghana, Africa: 1; Qatar: 1; London: 1; Australia: 1; Baku, Azerbaijan: 1; Spain: 1; Sweden: 1; Switzerland: 1; Taiwan: 1							Iran: 110; United States: 3; Canada: 3; Norway: 2; Turkey: 2; Brazil: 1; United Arab Emirates: 1; Qatar: 1; London: 1; Australia: 1; Spain: 1; Switzerland: 1						

Abbreviations: BSc, bachelor of science; DNP, doctorate of nursing practice; GP, general practitioner; MSc, master of science; PhD, doctor of philosophy; S, specialist; SubS, subspecialist.

**Table 2:** Delphi consensus process results regarding factors influencing toileting activities based on the ICF-CY.

	**Very high effect**	**High effect**	**Moderate effect**	**Low effect**	**No effect**	**Mean**	**Median**	**Mode**	**Percentiles**			**Agreement on effectiveness** ^a^	**The final result after the end of each round**
									**25**	**50**	**75**		
*Round 1*													
Body functions and structures													
1. Mental Function 1 (cognitive-perceptual functions including attention, memory, object recognition, visual perception, decision making, and planning and implementation of toileting activity steps)	79 (50.0%)	54 (34.2%)	21 (13.3%)	4 (2.5%)	0 (0.0%)	4.3165	4.5000	5.00	4.0000	4.5000	5.0000	84.2	Approved
2. Neuromusculoskeletal and movement-related functions (neuromuscular, gross, and fine motor functions)	74 (46.8%)	59 (37.3%)	22 (13.9%)	3 (1.9%)	0 (0.0%)	4.2911	4.0000	5.00	4.0000	4.0000	5.0000	84.1	Transferred to the 2nd round due to refinement
3. Genitourinary function (issues related to urology and the urinary tract)	64 (40.5%)	64 (40.5%)	24 (15.2%)	4 (2.5%)	2 (1.3%)	4.1646	4.0000	4.00^a^	4.0000	4.0000	5.0000	81.0	Approved
4. Sensory function (including touch function, proprioceptive function, and sensory functions related to temperature, pressure, etc.)	56 (35.4%)	71 (44.9%)	26 (16.5%)	5 (3.2%)	0 (0.0%)	4.1266	4.0000	4.00	4.0000	4.0000	5.0000	80.3	Transferred to the 2nd round due to refinement
5. Digestive function (issues related to the digestive system, bowel, and defecation.)	61 (38.6%)	62 (39.2%)	30 (19.0%)	5 (3.2%)	0 (0.0%)	4.1329	4.0000	4.00	4.0000	4.0000	5.0000	77.8	Approved
6. Mental Function 2 (psychological/behavioral functions and related problems including mood disorder, anxiety, depression, obsessive-compulsive disorders, behavioral disorders, such as stubbornness and fear)	54 (34.2%)	67 (42.4%)	29 (18.4%)	7 (4.4%)	1 (0.6%)	4.0506	4.0000	4.00	4.0000	4.0000	5.0000	76.6	Approved
7. Voice and speech function (for indicating the need for toileting, asking for help, etc.)	27 (17.1%)	53 (33.5%)	57 (36.1%)	18 (11.4%)	3 (1.9%)	3.5253	4.0000	3.00	3.0000	4.0000	4.0000	50.6	Transferred to the 2nd round due to rejection
Environment													
A. Products and technology													
1. Equipment and assistive devices used for toileting function	70 (44.3%)	62 (39.2%)	25 (15.8%)	1 (0.6%)	0 (0.0%)	4.2722	4.0000	5.00	4.0000	4.0000	5.0000	83.5	Approved
2. Information about equipment and assistive devices as well as teaching users how to use them	60 (38.0%)	71 (44.9%)	27 (17.1%)	0 (0.0%)	0 (0.0%)	4.2089	4.0000	4.00	4.0000	4.0000	5.0000	82.9	Approved
B. Natural environment and human-made changes													
1. Toilet environment (in terms of accessibility, cleanliness, adaptation of the toilet environment for disabled people, etc.)	84 (53.2%)	59 (37.3%)	12 (7.6%)	3 (1.9%)	0 (0.0%)	4.4177	5.0000	5.00	4.0000	5.0000	5.0000	90.5	Approved
2. Types of toilets (squat toilets, sitting toilets, or other types)	71 (44.9%)	53 (33.5%)	24 (15.2%)	10 (6.3%)	0 (0.0%)	4.1709	4.0000	5.00	4.0000	4.0000	5.0000	78.4	Approved
C. Support and relationships													
1. Amount and quality of child's/adolescent's communication with others	55 (34.8%)	75 (47.5%)	25 (15.8%)	3 (1.9%)	0 (0.0%)	4.1519	4.0000	4.00	4.0000	4.0000	5.0000	82.3	Approved
2. Receiving feedback from others regarding the correct or incorrect carrying out of the toileting steps	59 (37.3%)	71 (44.9%)	24 (15.2%)	4 (2.5%)	0 (0.0%)	4.1709	4.0000	4.00	4.0000	4.0000	5.0000	82.2	Approved
3. Amount and quality of others' communication with child/adolescent	56 (35.4%)	73 (46.2%)	25 (15.8%)	4 (2.5%)	0 (0.0%)	4.1456	4.0000	4.00	4.0000	4.0000	5.0000	81.6	Approved
4. Consequences of improper toileting repertoire, such as punishment	59 (37.3%)	70 (44.3%)	22 (13.9%)	3 (1.9%)	4 (2.5%)	4.1203	4.0000	4.00	4.0000	4.0000	5.0000	81.6	Approved
5. Receiving encouragement or reward	54 (34.2%)	71 (44.9%)	27 (17.1%)	6 (3.8%)	0 (0.0%)	4.0949	4.0000	4.00	4.0000	4.0000	5.0000	79.1	Approved
Personality traits of the main caregiver													
1. The caregiver's approach and reaction to the child's behavioral problems	73 (46.2%)	74 (46.8%)	11 (7.0%)	0 (0.0%)	0 (0.0%)	4.3924	4.0000	4.00	4.0000	4.0000	5.0000	93.0	Approved
2. The caregiver's level of knowledge and awareness about the child's readiness for toileting and how to teach toileting steps to the child	74 (46.8%)	66 (41.8%)	18 (11.4%)	0 (0.0%)	0 (0.0%)	4.3544	4.0000	5.00	4.0000	4.0000	5.0000	88.6	Transferred to the 2nd round due to refinement
3. Mood and personality characteristics of the caregiver	62 (39.2%)	75 (47.5%)	18 (11.4%)	3 (1.9%)	0 (0.0%)	4.2405	4.0000	4.00	4.0000	4.0000	5.0000	86.7	Approved
4. Type of care and child's/adolescent's relationship with the caregiver	58 (36.7%)	72 (45.6%)	27 (17.1%)	1 (0.6%)	0 (0.0%)	4.1835	4.0000	4.00	4.0000	4.0000	5.0000	82.3	Approved
5. The caregiver's level of expectations regarding toileting performance and his/her assessment of key aspects such as independence, safety, and efficiency	48 (30.4%)	78 (49.4%)	29 (18.4%)	3 (1.9%)	0 (0.0%)	4.0823	4.0000	4.00	4.0000	4.0000	5.0000	79.8	Approved
D. Attitudes toward toileting processes													
1. Individual attitudes of child/adolescence, family members, and others about toileting, carrying out the steps of toileting, menstrual management, etc.	58 (36.7%)	71 (44.9%)	24 (15.2%)	5 (3.2%)	0 (0.0%)	4.1519	4.0000	4.00	4.0000	4.0000	5.0000	81.6	Approved
2. Feeling embarrassed and ashamed of expressing problems related to bladder and bowel function, toileting, and menstruation by child/adolescence, family members, caregivers, health professionals, or other relevant people	58 (36.7%)	71 (44.9%)	23 (14.6%)	6 (3.8%)	0 (0.0%)	4.1456	4.0000	4.00	4.0000	4.0000	5.0000	81.6	Approved
3. Issues related to menstruation (such as providing the necessary training before period days, how to manage menstruation and comply with related health issues, restrictions on participation in period days such as prohibiting entry to places of pilgrimage such as mosques, shrines, and fire temples in some religious cultures by child/adolescence, family members, and others)	52 (32.9%)	63 (39.9%)	37 (23.4%)	5 (3.2%)	1 (0.6%)	4.0127	4.0000	4.00	3.0000	4.0000	5.0000	72.8	Transferred to the 2nd round due to rejection
4. How to carry out personal hygiene and clean oneself afterwards (such as the obligation to wash with water in other to preserve body cleanliness [Tahara] in some religious cultures by child/adolescence, family members, and others)	48 (30.4%)	64 (40.5%)	35 (22.2%)	8 (5.1%)	3 (1.9%)	3.9241	4.0000	4.00	3.0000	4.0000	5.0000	70.9	Transferred to the 2nd round due to rejection
5. How to perform the clothing manipulation before and after urination/defecation (e.g., taking off the clothes completely and putting them back on, pulling up the pants, or how to manage the clothes to prevent them from getting wet or contaminated. In order to maintain the cleanliness [Tahara] of the clothes in some religious cultures by child/adolescence, family members, and others)	53 (33.5%)	55 (34.8%)	40 (25.3%)	6 (3.8%)	4 (2.5%)	3.9304	4.0000	4.00	3.0000	4.0000	5.0000	68.3	Transferred to the 2nd round due to rejection
*Round 2*													
Body functions and structures													
1. Neuromusculoskeletal and movement-related function (effect of neuromuscular functions, gross and fine motor functions, motor control and coordination, and praxis on toileting function)	53 (41.7%)	67 (52.8%)	5 (3.9%)	2 (1.6%)	0 (0.0%)	4.3465	4.0000	4.00	4.0000	4.0000	5.0000	94.5	Approved
2. Sensory functions (such as touch function, smell function, proprioception, sensory processing and integration, interoception, vestibular, sensory function related to temperature, pressure, and other stimuli).	68 (53.5%)	50 (39.4%)	7 (5.5%)	2 (1.6%)	0 (0.0%)	4.4488	5.0000	5.00	4.0000	5.0000	5.0000	92.9	Approved
3. Feeling pain and burning sensation before, during, or after urination and defecation in toileting function	49 (38.6%)	59 (46.5%)	15 (11.8%)	4 (3.1%)	0 (0.0%)	4.2047	4.0000	4.00	4.0000	4.0000	5.0000	85.1	Approved
4. Voice and speech function (the influence of voice and speech function on the execution of toilet-related activities, such as announcing the need to use the toilet and requesting assistance from a caregiver or others)	42 (33.1%)	54 (42.5%)	28 (22.0%)	3 (2.4%)	0 (0.0%)	4.0630	4.0000	4.00	4.0000	4.0000	5.0000	75.6	Approved
Environment													
A. Products and technology													
1. Medications and their side effects	54 (42.5%)	58 (45.7%)	11 (8.7%)	4 (3.1%)	0 (0.0%)	4.2756	4.0000	4.00	4.0000	4.0000	5.0000	88.2	Approved
C. Support and relationship (personality traits of the main caregiver)													
1. Main caregiver's level of awareness of the child's readiness for toilet training, the method of teaching this skill to the child, the generalization of the strategies provided by the treatment team to home or other environments, and their follow-up on toileting performance	69 (54.3%)	53 (41.7%)	4 (3.1%)	1 (0.8%)	0 (0.0%)	4.4961	5.0000	5.00	4.0000	5.0000	5.0000	96.0	Approved
2. Main caregiver's economic status and ability to afford the cost of purchasing necessary equipment and assistive devices for toileting performance	53 (41.7%)	63 (49.6%)	11 (8.7%)	0 (0.0%)	0 (0.0%)	4.3307	4.0000	4.00	4.0000	4.0000	5.0000	91.3	Approved
3. Main caregiver's physical ability to perform the toileting steps or assist the individual in performing the steps	61 (48.0%)	54 (42.5%)	9 (7.1%)	3 (2.4%)	0 (0.0%)	4.3622	4.0000	5.00	4.0000	4.0000	5.0000	90.5	Approved
D. Attitudes toward toileting processes													
1. The influence of personal beliefs and attitudes, as well as societal attitudes, on issues related to menstruation (such as providing necessary education prior to menstruation, managing menstruation, and adhering to health-related issues and participation restrictions during menstruation in some religious cultures such as prohibiting entry to places of worship such as mosques, shrines, and fire temples) in some religious cultures	54 (42.5%)	64 (50.4%)	5 (3.9%)	4 (3.1%)	0 (0.0%)	4.3228	4.0000	4.00	4.0000	4.0000	5.0000	92.9	Approved
2. The influence of individual beliefs and attitudes, as well as societal attitudes, on the implementation of health and hygiene practices (such as the requirement of washing with water for the sake of bodily purity in some religious cultures [Tahara])	66 (52.0%)	44 (34.6%)	13 (10.2%)	4 (3.1%)	0 (0.0%)	4.3543	5.0000	5.00	4.0000	5.0000	5.0000	86.6	Approved
3. The influence of individual beliefs and attitudes, as well as the prevailing societal attitudes, on the way the clothing management is executed (e.g., completely removing clothes and rewearing them, pulling up trousers, or managing clothing to prevent them from getting wet or dirty, etc., to maintain the purity of clothing [Tahara] in some religious cultures)	58 (45.7%)	52 (40.9%)	13 (10.2%)	4 (3.1%)	0 (0.0%)	4.2913	4.0000	5.00	4.0000	4.0000	5.0000	86.6	Approved
4. Not disseminating related materials in simple language about toilet training in public media such as radio, television, and newspapers to increase public awareness of toileting performance	60 (47.2%)	50 (39.4%)	15 (11.8%)	2 (1.6%)	0 (0.0%)	4.3228	4.0000	5.00	4.0000	4.0000	5.0000	86.6	Approved
Personal factors that influence the carrying out toileting function													
1. Presence of diseases associated with CP	56 (44.1%)	63 (49.6%)	7 (5.5%)	1 (0.8%)	0 (0.0%)	4.3701	4.0000	4.00	4.0000	4.0000	5.0000	93.7	Approved
2. Age	66 (52.0%)	49 (38.6%)	10 (7.9%)	2 (1.6%)	0 (0.0%)	4.4094	5.0000	5.00	4.0000	5.0000	5.0000	90.6	Approved
3. Personal habits (such as nutritional habits and urination and defecation habits)	45 (35.4%)	61 (48.0%)	20 (15.7%)	1 (0.8%)	0 (0.0%)	4.1811	4.0000	4.00	4.0000	4.0000	5.0000	83.4	Approved
4. Education level	40 (31.5%)	62 (48.8%)	21 (16.5%)	4 (3.1%)	0 (0.0%)	4.0866	4.0000	4.00	4.0000	4.0000	5.0000	80.3	Approved
5. Gender	40 (31.5%)	57 (44.9%)	18 (14.2%)	10 (7.9%)	2 (1.6%)	3.9685	4.0000	4.00	4.0000	4.0000	5.0000	76.4	Approved

^a^Agreement on effectiveness: very high effect percentile+high effect percentile.

## Data Availability

The data used in this study is available upon reasonable request to the corresponding author. Due to privacy and ethical considerations, some restrictions may apply to the availability of sensitive or confidential data.

## Supporting Information

Supporting Information 1 File S1: search strategy.

Supporting Information 2 File S2: Delphi questionnaire—Round 1.

Supporting Information 3 File S3: Delphi questionnaire—Round 2.
