# Supplementary material for: A Delphi Exploration of Toileting Activity Performance in Individuals With Cerebral Palsy Within the ICF-CY Framework: Unveiling Influential Factors
Source: Occup Ther Int. 2024 Oct 7;2024:9994862. doi: 10.1155/2024/9994862 (PMC11473173; doi:10.1155/2024/9994862)
Supplement: Supporting Information 1 — File S1: search strategy. [file 9994862.f1.docx]

**Appendix A**

***Search strategy***

Two authors asynchronously reviewed the articles related to the subject of study between 1990 and 2022 in English and Persian languages using the following keywords in Google Scholar, PubMed, Medline, ERIC, PsycINFO, OVID, ProQuest databases, CINAHL, Cochrane library, Scopus, Web of Knowledge, OTseeker and Rehadat.

- Cerebral palsy
- Toileting, Toilet*
- Function, Dysfunction, Activity, Ability, Performance
- Influential factors
- Toilet training
- Training, Education
- Evaluation, Assessment, Intervention
- Tool, Scale, Questionnaire, Inventory
- Bladder, Bowel, Urination, Micturition, Defecation, Urinary, Fecal, Incontinence, Control
- Menstruation
- Activities of Daily Livings (ADL)
- International Classification of Functioning, Disability, and Health for Children and Youth (ICF-CY) framework
- Body functions and structures, Sensory-motor, Cognitive-Perceptual, Psychosocial, Behavioral, Digestive, Voice and speech
- Support and relationships
- Main caregiver
- Participation, participation restrictions
- Taboo, Attitude, Culture, Religion
- Environment
- Toilet assistive device, Products and technology
