# Supplementary material for: A Delphi Exploration of Toileting Activity Performance in Individuals With Cerebral Palsy Within the ICF-CY Framework: Unveiling Influential Factors
Source: Occup Ther Int. 2024 Oct 7;2024:9994862. doi: 10.1155/2024/9994862 (PMC11473173; doi:10.1155/2024/9994862)
Supplement: Supporting Information 2 — File S2: Delphi questionnaire—Round 1. [file 9994862.f2.docx]

***Appendix B: Delphi Questionnaire- Round 1***

**Dear Professor/Colleague,**

Members of the research team intend to explore the factors that influence toileting activity performance in individuals with cerebral palsy.

By reviewing literature and implementing other phases such as focus groups, the factors that deem to influence toileting were collected and classified based on the ICF-CY model.

In the following, there are two sections. Section A asks some questions about your background. In section B, factors that might influence each step of the toileting function processes will be presented and you will be asked to identify the level of their influence on toileting in a five levels scale.

Your participation in this research will surely help to explore influential factors and shed light on this important function.

**Section A: Participant background**

Please fill in your personal information in the space below and answer the questions.

**Demographic information regarding experts**

- First name:
- Last name:
- Age in a year:
- Gender:
- Country of residence:
- University (if you work at a university):
- Major:
- Education level:

Bachelor 🗆 Master 🗆 PhD and above 🗆 General practitioner 🗆	Specialist physician 🗆	subspecialist physician 🗆

- Duration of experience in your field in year:
- In general, how many years of work experience do you have in the following fields? (please indicate 0 if having no experience)
- Educational activities:
- Clinical activities:
- Executive activities:
- Duration of work experience in the field of medical treatment or rehabilitation of patients with cerebral palsy in year:
- Are disorders in the field of urinary/fecal incontinence or toileting behavior of patients, a concern for you, the patients or the patients' families?

Yes 🗆	No 🗆

- In your field of work, to what extent do you provide toilet training/ intervention to your clients or treat disorders related to it?

Very high 🗆	High 🗆	Medium 🗆	A little 🗆	a very little 🗆

**Section B: Factors influencing the processes of toileting function**

The research team encountered some components and factors that influence the toileting function. Hence, we have collected a set of these components and factors based on the ICF-CY model and in the form of personal and environmental factors. In this section, please tick the box that you think is appropriate for each factor.

| **Very high effect**  **(5)** | **High**  **effect**  **(4)** | **Moderate effect**  **(3)** | | | **Low**  **effect**  **(2)** | | | **No**  **effect**  **(1)** |  |
| --- | --- | --- | --- | --- | --- | --- | --- | --- | --- |
| - **Section B:Factors influencing the carrying out toileting function** | | | | | | | | | |
| **Body Functions and structures** | | | | | | | | | |
| A. **Mental Functions** | | | | | | | | | |
| - Global mental functions (Consciousness functions, Orientation functions, Global psychosocial functions, Temperament and personality functions, …) - Specific mental functions (Attention functions, Memory functions, Emotional functions, Perceptual function, Basic and Higher-level cognitive function, Mental functions of language, …) | | | | | | | | | |
| 5 | 4 | 3 | | | 2 | | | 1 | 1. Impairments in cognitive/perceptual functions related to the processes of toileting function (including defects in attention, memory, object recognition, visual perception, touch perception, decision making, planning and implementation of activity steps, sequence, problem solving, production and receiving verbal and non-verbal messages, etc.) |
| 5 | 4 | 3 | | | 2 | | | 1 | 2. Psychological/behavioral problems (including mood, anxiety, depression, and obsessive-compulsive disorders, behavioral disorders such as stubbornness, fear, etc.) |
| B. **Sensory Functions and pain** | | | | | | | | | |
| 5 | 4 | 3 | | | 2 | | | 1 | Impairments in sensory functions related to the carrying out toileting steps (including problems in touch function, proprioceptive function, sensory functions related to temperature, pressure, etc.) |
| C. **Voice and speech functions** | | | | | | | | | |
| 5 | 4 | 3 | | | 2 | | | 1 | Audio-speech impairments that prevent the child from indicating the need for toileting, asking for help, etc. |
| D. **Functions of the digestive, metabolic and endocrine systems** | | | | | | | | | |
| 5 | 4 | | 3 | | 2 | | | 1 | Digestive and defecation impairments |
| E. **Genitourinary and reproductive functions** | | | | | | | | | |
| 5 | 4 | | | 3 | 2 | | 1 | | Urological impairments and defects of the urinary tract |
| **F. Neuromusculoskeletal and movement-related functions** | | | | | | | | | |
| 5 | 4 | | 3 | | 2 | 1 | | | Nneuromuscular, gross and fine motor functions impairments |
| **Environment** | | | | | | | | | |
| **A. Products and technology** | | | | | | | | | |
| 5 | 4 | 3 | | | 2 | | | 1 | 1. 1. Equipment and assistive devices used for toileting function |
| 5 | 4 | 3 | | | 2 | | | 1 | 2. Providing information about equipment and assistive devices as well as teaching users how to use them |
| **B. Natural environment and human-made changes** | | | | | | | | | |
| 5 | 4 | 3 | | | 2 | | | 1 | 1. Toilet environment (indoor and outdoor including school, workplace and other social environments) in terms of accessibility, cleanliness, adaptation of the toilet environment for disabled people, etc. in offices, schools, universities, parks and other public places in the city. |
| 5 | 4 | 3 | | | 2 | | | 1 | 2. Different types of toilet (Squat toilets, sitting toilets or other types) |
| **C. Support and relationships** | | | | | | | | | |
| 5 | 4 | 3 | | | 2 | | | 1 | 1. The amount and quality of communication between family members, relatives, friends, classmates, colleagues, health professionals and other professionals, neighbors, etc. with child/adolescent, in other to carrying out the steps of toileting, menstrual management training, etc. |
| 5 | 4 | 3 | | | 2 | | | 1 | 2. The amount and quality of child's/adolescent's communication with family members, relatives, friends, classmates, colleagues, health professionals and other professionals, neighbors, etc. to express issues and problems related to bowel and bladder function or dysfunction, carrying out the steps of toileting, menstrual management, etc. |
| 5 | 4 | 3 | | | 2 | | | 1 | 3. The feedback that the child/adolescent receives from the family members/caregiver/group of friends/classmates/colleagues/relatives and other people regarding the correct or incorrect carrying out of the toileting steps. |
| 5 | 4 | 3 | | | 2 | | | 1 | 4. Receiving encouragement or reward if mistakes are not repeated during the toileting function, or making progress while doing it. |
| 5 | 4 | 3 | | | 2 | | | 1 | 5. Consequences of improper toileting repertoire, such as verbal punishment, corporal punishment, expulsion from a game or classroom, rejection from friends, etc. |
| - **Personality traits of the main caregiver** | | | | | | | | | |
| 5 | 4 | 3 | | | 2 | | | 1 | 1. Type of care and child's/adolescent's relationship with the caregiver (mother, father, nurse, or other caregivers) |
| 5 | 4 | 3 | | | 2 | | | 1 | 2. Mood and personality characteristics of the caregiver (such as disorders like anxiety disorders, depression, obsession, etc.) |
| 5 | 4 | 3 | | | 2 | | | 1 | 3. The caregiver's level of knowledge and awareness about the child's readiness for toileting and how to teach toileting steps to the child |
| 5 | 4 | 3 | | | 2 | | | 1 | 4. The caregiver's approach and reaction to the child's behavioral problems (such as stubbornness, fear, playfulness, refusal to go to the toilet, toileting in a place other than the toilet, etc.) |
| 5 | 4 | 3 | | | 2 | | | 1 | 5. The caregiver's level of expectations regarding toileting performance and their assessment of key aspects related to the carrying out toileting procedures such as independence/efficiency/safety. |
| **D. Attitudes toward toileting processes** | | | | | | | | | |
| - Individual attitudes of family members, caregivers, relatives, friends, classmates, colleagues, health professionals and other professionals, neighbors and the others. - Attitudes of the society, ideologies, rules and the governing norms of the society, cultural attitudes, religious/spiritual tendencies city or country of residence. | | | | | | | | | |
| 5 | 4 | 3 | | | 2 | | | 1 | 1. Individual attitudes of child/adolescence, family members, caregivers, relatives, friends, classmates, colleagues, health professionals and other professionals, neighbors and the others about toileting, carrying out the steps of toileting, menstrual management, etc. |
| 5 | 4 | 3 | | | 2 | | | 1 | 2. Feeling embarrassed and ashamed of expressing problems related to bladder and bowel function, toileting and menstruation by child/adolescence, family members, caregivers, health professionals or other relevant people. |
| 5 | 4 | 3 | | | 2 | | | 1 | 3. How to carry out personal hygiene and cleaning oneself afterwards (such as the obligation to wash with water in other to preserve body cleanliness (Tahara) in some religious cultures) by child/adolescence, family members, caregivers, health professionals or other relevant people. |
| 5 | 4 | 3 | | | 2 | | | 1 | 4. How to perform the clothing manipulation before and after urination (for example, taking off the clothes completely and putting them back on, pulling up the pants, or how to manage the clothes to prevent them from getting wet or contaminated, etc. In order to maintain the cleanliness (Tahara) of the clothes in some religious cultures) by child/adolescence, family members, caregivers, health professionals or other relevant people. |
| 5 | 4 | 3 | | | 2 | | | 1 | 5. Issues related to menstruation (such as providing the necessary training before period days, how to manage menstruation and comply with related health issues, restrictions on participation in period days (such as prohibiting entry to places of pilgrimage such as mosques, shrines, fire temples, etc. in some religious cultures)) by child/adolescence, family members, caregivers, health professionals or other relevant people in the society. |
| In this section, please mention other factors that you think affect the carrying out toileting behavior and are not mentioned in this list. | | | | | | | | | |
