# Supplementary material for: A Delphi Exploration of Toileting Activity Performance in Individuals With Cerebral Palsy Within the ICF-CY Framework: Unveiling Influential Factors
Source: Occup Ther Int. 2024 Oct 7;2024:9994862. doi: 10.1155/2024/9994862 (PMC11473173; doi:10.1155/2024/9994862)
Supplement: Supporting Information 3 — File S3: Delphi questionnaire—Round 2. [file 9994862.f3.docx]

***Appendix C: Delphi Questionnaire- Round 2***

***International Delphi study: Round 2***

***(Taking about 15 minutes to be completed)***

Dear Professor/Colleague,

We would like to express our sincere gratitude for your participation in the first round of our study and for sharing your valuable opinions. Our study aimed to explore the "factors that influence toileting activity performance in individuals with cerebral palsy."

We have carefully reviewed all the responses received, and after analyzing the results, we found that there was a lack of 75% agreement on some items in the first round, and other items were added by the participants. Therefore, we believe that it is necessary to conduct a second round of this Delphi study.

We kindly request your assistance in expressing your helpful comments on the items presented in this second round. We would greatly appreciate it if you could rate the items using the Likert scale and state your reason for choosing the option. Your answer will help us in the implementation of this study.

The items in this round are presented in two sections and it will take about 15 minutes to be completed:

Section A) contains items from the first round that require further evaluation and input from the participants. The percentage of agreement obtained related to these items in the first round was as follows:

| 1. The influence of voice and speech function on the execution of toilet-related activities, such as announcing the need to use the toilet and requesting assistance from a caregiver or others. | **50/6 %** |
| --- | --- |
| 2. The influence of individual beliefs and attitudes, as well as societal attitudes, on the implementation of health and hygiene practices. | **70/9 %** |
| 3. The influence of individual beliefs and attitudes, as well as the prevailing societal attitudes, on the way the clothing management is executed. | **68/4 %** |
| 4. The influence of personal beliefs and attitudes, as well as societal attitudes, on issues related to menstruation. | **72/8 %** |

Section B) contains other items that were added by the participants as factors that influence carrying out toileting function.

Thank you for your continued support in this important research endeavor.

**Section A: Items from the first round and need to be re-ranked**

| **Very­ high effect**  **(5)** | **High effect**  **(4)** | **Moderate effect**  **(3)** | **Low**  **effect**  **(2)** | **No**  **effect**  **(1)** | - **Factors influencing the carrying out toileting function** |
| --- | --- | --- | --- | --- | --- |
| 5 | 4 | 3 | 2 | 1 | 1. The influence of voice and speech function on the execution of toilet-related activities, such as announcing the need to use the toilet and requesting assistance from a caregiver or others.  - Reason for choosing the option: |
| 5 | 4 | 3 | 2 | 1 | 2. The influence of individual beliefs and attitudes, as well as societal attitudes, on the implementation of health and hygiene practices (such as the requirement of washing with water for the sake of bodily purity in some religious cultures (Tahara)).  - Reason for choosing the option: |
| 5 | 4 | 3 | 2 | 1 | 3. The influence of individual beliefs and attitudes, as well as the prevailing societal attitudes, on the way the clothing management is executed (for example, completely removing clothes and re-wearing them, pulling up trousers, or managing clothing to prevent them from getting wet or dirty, etc., to maintain the purity of clothing (Tahara) in some religious cultures).  - Reason for choosing the option: |
| 5 | 4 | 3 | 2 | 1 | 4. The influence of personal beliefs and attitudes, as well as societal attitudes, on issues related to menstruation (such as providing necessary education prior to menstruation, managing menstruation and adhering to health-related issues, and participation restrictions during menstruation in some religious cultures such as prohibiting entry to places of worship such as mosques, shrines, fire temples, etc.) in some religious cultures.  - Reason for choosing the option: |

- **Second B: Other items that were added by the participants**

| **Very high effect**  **(5)** | **High effect**  **(4)** | **Moderate effect**  **(3)** | **Low**  **effect**  **(2)** | **No**  **effect**  **(1)** | - **Other factors influencing the carrying out toileting function** |
| --- | --- | --- | --- | --- | --- |
| 5 | 4 | 3 | 2 | 1 | 1. The degree of effect of sensory functions related to the execution of toileting stages (such as Touch function, Smell function, Taste function, proprioception, sensory processing and integration, interoception, vestibular, sensory function related to temperature, pressure, and other stimuli).  - Reason for choosing the option: |
| 5 | 4 | 3 | 2 | 1 | 2. The degree of effect of feeling pain and burning sensation before, during, or after urination and defecation on toileting function  - Reason for choosing the option: |
| 5 | 4 | 3 | 2 | 1 | 3. The degree of effect of neuromuscular functions, gross and fine motor functions, motor control and coordination, and praxis on toileting function.  - Reason for choosing the option: |
| 5 | 4 | 3 | 2 | 1 | 4. The degree of effect of the consumed medications and their side effects on toileting function.  - Reason for choosing the option: |
| 5 | 4 | 3 | 2 | 1 | 5. The degree of effect of the main caregiver's physical ability to perform the toileting steps or assist the individual in performing the steps.  - Reason for choosing the option: |
| 5 | 4 | 3 | 2 | 1 | 6. The degree of effect of the main caregiver's economic status and ability to afford the cost of purchasing necessary equipment and assistive devices on toileting performance.  - Reason for choosing the option: |
| 5 | 4 | 3 | 2 | 1 | 7. The degree of effect of the caregiver's level of cognition and awareness of the child's readiness for toilet training, the method of teaching this skill to the child, the generalization of the strategies provided by the treatment team to home or other environments, and their follow-up on toileting performance.  - Reason for choosing the option: |
| 5 | 4 | 3 | 2 | 1 | 8. The degree of effect of not disseminating related materials in simple language about toilet training in public media such as radio, television, and newspapers to increase public awareness on toileting performance.  - Reason for choosing the option: |
| - **Personal factors that influence the carrying out toileting function:** | | | | | |
| 5 | 4 | 3 | 2 | 1 | 9. Age  - Reason for choosing the option: |
| 5 | 4 | 3 | 2 | 1 | 10. Gender  - Reason for choosing the option: |
| 5 | 4 | 3 | 2 | 1 | 11. Presence of diseases associated with cerebral palsy  - Reason for choosing the option: |
| 5 | 4 | 3 | 2 | 1 | 12. Personal habits (such as nutritional habits, urination and defecation habits, etc.)  - Reason for choosing the option: |
| 5 | 4 | 3 | 2 | 1 | 13. Education level  - Reason for choosing the option: |
| - In this section, please mention any other comments or suggestions you have. | | | | | |
